# Reference Values for Permanent-Tooth Emergence in Hungarian Children: A Cross-Sectional Study

**DOI:** 10.3390/dj13110542

**Published:** 2025-11-18

**Authors:** Zsuzsa Kapusi-Papp, János Máth, Judit Ágnes Nemes

**Affiliations:** 1 Department of Pediatric and Preventive Dentistry, Faculty of Dentistry, University of Debrecen, 4032 Debrecen, Hungary; pappzsuzsa@dental.unideb.hu; 2 Institute of Psychology, Faculty of Humanities, University of Debrecen, 4032 Debrecen, Hungary; math.janos@arts.unideb.hu

**Keywords:** tooth eruption, permanent teeth, eruption sequence, emergence time, Hungary

## Abstract

**Background/Objectives**: Population-specific data on permanent-tooth eruption are essential for accurate diagnosis, treatment planning, and public health strategies. In Hungary, current clinical practice relies on outdated international eruption standards. The aim of this study was to determine the timing and sequence of permanent tooth emergence in Hungarian children and provide sex-specific eruption standards. **Methods**: A cross-sectional study was conducted based on dental screening records of 2948 children aged 4–15 years in Debrecen, Hungary, during the 2015–2016 school year. Probit regression was used to estimate median eruption times and percentiles, and eruption sequences were assessed by sex and jaw. **Results**: Girls exhibited earlier eruption than boys for all teeth except mandibular central incisors (difference: 1.9–8.9 months; *p* < 0.05). Mandibular teeth generally preceded maxillary teeth, though premolars in boys showed the opposite trend. Eruption sequences varied by sex, particularly in the canine-premolar region. The 5th-95th percentile eruption range was widest for second molars (4.8 years) and narrowest for maxillary central incisors (2.3 years). **Conclusions**: This study provides the first modern eruption standards for Hungarian children. These sex-specific reference values are clinically useful for assessing delayed eruption, guiding radiographic decisions, and optimizing the timing of preventive and orthodontic interventions.

## 1. Introduction

Permanent-tooth eruption is a complex and tightly regulated developmental process comprising both active and passive phases. The active phase involves the axial movement of a tooth from its intraosseous position into its functional occlusion within the dental arch, while the passive phase includes apical migration of the epithelial attachment and compensatory movements that maintain occlusal contact throughout life. Tooth emergence—defined as the moment a tooth breaks through the gingiva and becomes visible in the oral cavity—represents only one stage of this process, and concludes when the tooth reaches occlusion with its antagonist [1].

Genetic and environmental determinants jointly regulate eruption timing. While sex, race/ethnicity, and craniofacial morphology contribute to genetically mediated variation [2,3,4,5], environmental influences such as nutrition, body mass index (BMI) [6,7], socioeconomic status [8,9], caries in primary predecessors [10,11], and secular trends [12] significantly modulate eruption. Systemic diseases and syndromes can further disrupt this process [13,14]. Twin and family studies confirm a heritable component, although the specific genetic variants and their effect sizes remain incompletely characterized [2].

Population studies consistently demonstrate small but measurable intergroup differences. Beyond genetic and racial variation, environmental and socioeconomic contexts also influence eruption timing. Epidemiological studies have shown that urban children generally experience earlier tooth emergence than their rural counterparts [10,15], primarily due to differences in nutrition, healthcare access, and living standards. Urban environments are associated with higher dietary quality and greater availability of fortified foods, whereas undernutrition and limited access to preventive care in rural areas can delay eruption. These contextual disparities highlight the importance of establishing regional reference data rather than relying solely on aggregated national or international standards.

Systematic reviews and cross-sectional analyses indicate statistically significant variation in eruption timing between populations, typically in the order of several months and influenced by tooth type and sex [16,17]. Earlier eruption has been observed in certain African-ancestry and Indigenous populations compared with European-ancestry cohorts, though findings are heterogeneous [18,19]. Notably, sex differences—girls’ teeth erupting earlier than boys—are more consistent and of greater magnitude than most race-related effects.

Population-specific standards are essential for clinical and public health applications. While eruption sequences show broad cross-national similarities, reliance on outdated or non-local reference data risks misclassification and suboptimal treatment planning.

In Hungary, current practice often relies on decades-old Anglo-Saxon data [20,21], which may not reflect contemporary patterns, therefore our aim was to determine the timing and sequence of permanent-tooth emergence in a large, representative cohort of children in Debrecen and its surrounding region. Based on our clinical experience, an increasing number of children already present with erupted permanent teeth at school entry (around age six). For this reason, the present investigation was extended to include preschool children over the age of four, in order to capture earlier phases of eruption and provide a more comprehensive dataset.

This study aimed to (1) determine sex-specific median ages and percentile distributions for the eruption of each permanent tooth, (2) describe the typical sequence of emergence in Hungarian children, and (3) compare these findings with international reference data. We hypothesized that Hungarian children would exhibit slightly earlier eruption patterns than those reported in historical Anglo-Saxon datasets, reflecting contemporary changes in growth and development.

## 2. Methods

### 2.1. Study Setting

Debrecen, the second most populous city in Hungary, is located approximately 220 km east of Budapest. In 2016, it had a population of 202,520. The city has a relatively homogeneous ethnic composition, with non-Caucasian residents making up less than 1% of the permanent population. Debrecen and its surrounding areas are served by 43 kindergartens and 38 primary schools.

In Hungary, based on Ministerial Decree 26/1997 (IX.3) on school health services, all children aged 3 to 18 enrolled in organized education are required to undergo annual school dental examinations. In Debrecen, these examinations are conducted by trained specialists with the written consent of the children’s legal guardians. The assessments take place in school dental offices using a dental chair, dental mirror, and dental probe under appropriate lighting conditions. During the examinations, complete dental status is recorded. In addition to documenting decayed, missing, and filled teeth, both healthy primary and permanent teeth are recorded. Fully erupted teeth are marked with a dot, while partially erupted teeth—defined as those with any portion of the crown visible through the gingiva—are marked with a wavy line. Occlusion, gingival and periodontal status, oral hygiene, and any developmental abnormalities of the teeth, soft tissues, or jaws are also documented. Radiographic imaging is not performed during group screening sessions.

### 2.2. Ethics

Written informed consent was obtained from the parents/legal guardians of all the examined children prior to the school dental screening examinations. The study was conducted in accordance with the Declaration of Helsinki and approved by the Regional and Institutional Research Ethics Committee of the University of Debrecen (permit number: 5613-2020; date of approval: 17 December 2020).

### 2.3. Sample Population

For this cross-sectional study, we retrospectively reviewed the dental records of 3132 children examined during the 2015–2016 academic year in two randomly selected kindergartens and eight elementary schools in Debrecen.

The study included children aged between 4 and 15 years. Clinical experience indicates that a substantial proportion of children starting school at 6 years already have permanent teeth, typically the first molars and lower central incisors. Excluding 4- and 5-year-old children would omit early eruptions, potentially biasing the eruption curves and compromising the accuracy of the statistical analyses. Previous international studies support this approach; for instance, Shaweesh [22] reported that 5% of first molars in girls erupt between ages 4 and 5, while the earliest 5% of lower and upper central incisors emerge between 5 and 6 years. Similar findings were documented by Rajić [23], Altamoaitiene [24], Šindelářová [25], and Moslemi [26]. To ensure inclusion of subjects with late-erupting teeth, the upper age limit was set at 15 years.

Children who had a complete dental chart from the 2015–2016 school year were included, and children were not excluded based on systemic diseases, physical or mental disabilities, nationality, socioeconomic status, religious affiliation or birthplace. A total of 175 children were excluded due to age (younger than 4 years or older than 15 years), and 9 were excluded due to non-Caucasian origin. The final study sample included 2948 children (1487 boys and 1461 girls), representing approximately 11.8% of the 4–15-year-old population of Debrecen in 2016.

### 2.4. Data Collection

Dental chart data were transferred to a Microsoft Excel 2016 (v16.0) spreadsheet. Each entry was anonymized using a unique identifier generated from the first letter of the educational institution and the sequence number of the screening. Additional data included the child’s sex, and date of birth, and the date of their examination. Chronological age was calculated as the difference between the date of birth and the date of examination, and individuals were grouped into one-month age intervals based on total months lived.

For each permanent tooth, eruption status was recorded: teeth marked in the chart with either a dot (fully erupted) or a wavy line (partially erupted) were classified as “present” and coded as 1; unerupted teeth were coded as 0. Teeth extracted for orthodontic or known reasons were also considered “present.” If a tooth was absent and suspected to be congenitally missing or impacted, it was excluded from the analysis. Third molars were excluded entirely due to their highly variable eruption patterns and a global agenesis prevalence exceeding 20% [27].

### 2.5. Statistical Analysis

Statistical analysis was performed using SPSS software (Version 25.0). The timing and sequence of permanent-tooth emergence were estimated by applying probit regression to cross-sectional data. The presence or absence of each permanent tooth in each subject was coded as a binary outcome (0/1), and age (in months) was used as the explanatory (predictor) variable. From the fitted probit function, one can derive percentile values, in particular the median age of eruption (i.e., the age at which the model predicts a 50% probability of eruption for that tooth). Using these median (and other percentile) estimates across all tooth types, it is possible to infer the relative order (chronology) of eruption events at the population level. This approach is justified and has precedent in the dental literature. Heidmann (1986) compared graphical, arithmetic, and probit procedures for estimating the median age of eruption, and found that probit regression provides more reliable and unbiased estimators under many realistic sampling conditions and age distributions [28]. Its use is especially practical when longitudinal follow-up is not feasible.

We acknowledge that individual-level variability—due to local, systemic, genetic, or environmental factors—may lead to deviations from the “population-average” sequence. Therefore, we interpret our findings as describing average emergence chronology and timing in our sample population, not as strict deterministic sequences at the individual level. Nonetheless, probit-based percentile estimates from cross-sectional data provide a well-accepted and statistically sound method for comparing eruption timing and relative order across teeth in epidemiological and dental growth studies.

To determine whether significant asymmetry existed between right- and left-side eruption times, we conducted a preliminary comparison. As differences for all teeth were under 0.05% and showed no systematic pattern, data from both sides were pooled for analysis.

The median emergence ages and the 5th, 25th, 75th, and 95th percentiles were calculated for each tooth, using 95% confidence intervals (CI). The significance level was set at the 0.05 level. Analyses were conducted for the total sample and stratified by sex, and final emergence ages were converted from months to years for presentation.

Eruption sequences were established based on the median emergence ages and associated 95% confidence intervals, differentiated by jaw and sex. Differences between median eruption times were considered statistically significant when their confidence intervals did not overlap. Emergence curves were generated to illustrate the dynamics and probability of tooth eruption over time.

## 3. Results

Preliminary analyses revealed no significant differences in emergence times between the right and left sides; therefore, data from both sides were pooled for all subsequent analyses. The median ages for the emergence of maxillary and mandibular permanent teeth, stratified by sex, are presented in Table 1.

**Figure 1:**
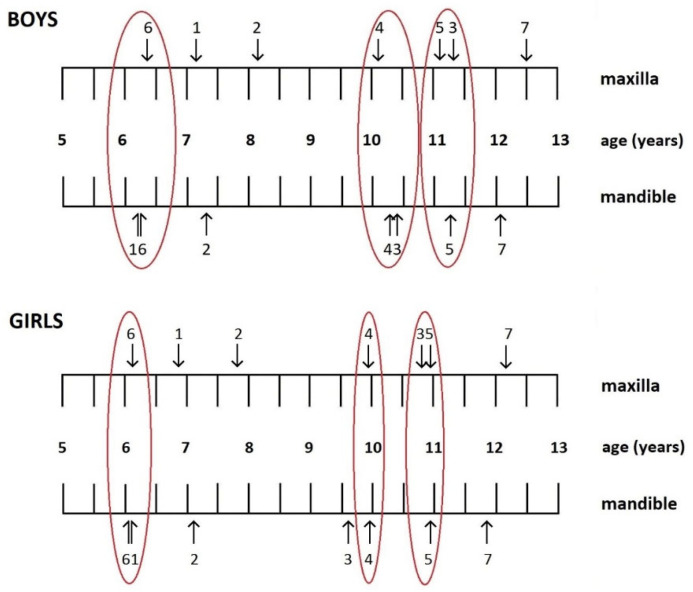
Sequence of permanent tooth eruption in boys and girls.

The sequence of tooth emergence differed between sexes (Figure 1). In girls, eruption began with the mandibular first molars, while in boys, it started with the mandibular central incisors. Among girls, the mandibular canines erupted before the first premolars in the lower jaw and the second premolars in the upper jaw. In contrast, among boys, maxillary canines preceded both premolars, but in the mandible, the canines erupted after the first premolars.

There is no significant difference between the eruption times of teeth in the same circle.

When considering the 95% confidence intervals, some teeth showed overlapping eruption windows, indicating near-simultaneous emergence. In both sexes, the mandibular central incisors and the first molars (both arches) emerged almost simultaneously. Similarly, the upper canines and second premolars also shared overlapping intervals. In boys, the first premolars (maxillary and mandibular) and the mandibular canines erupted concurrently, while in girls, the upper and lower first premolars emerged with negligible timing differences.

Table 2 presents the 5th, 25th, 50th (median), 75th, and 95th percentiles for each permanent tooth, overall and by sex. In 5% of children, the first permanent teeth (mandibular first molars and central incisors, maxillary first molars) emerged as early as 4.7–4.8 years of age. Even among children with late-erupting teeth, these teeth had emerged into the oral cavity by 7.5 years. At the other end of the timeline, in 95% of children their final permanent teeth (maxillary second molars) had erupted by 14.7 years, whereas full eruption had occurred in 5% (excluding third molars) by age 10.

**Figure 2:**
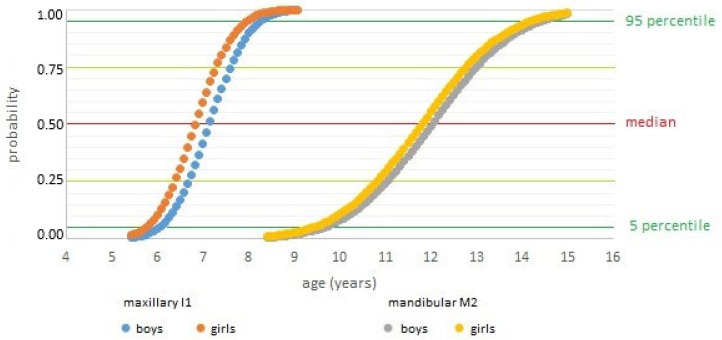
Emergence curves for the maxillary central incisors and the mandibular second molars. I1—central incisor; M2—second molar.

Examining the dynamics of eruption (Figure 2), the mandibular second molars showed the longest eruption interval (4.8 years between the 5th and 95th percentiles), while the maxillary central incisors erupted over the shortest period (2.3 years). The most intense phase of emergence—between the 25th and 75th percentiles—accounted for roughly 40% of the total eruption span. This interval lasted approximately one year for all incisors and first molars, and around two years for other teeth.

The total duration of emergence (5th–95th percentile range) can be approximated as the interquartile range ±1 year. However, for teeth with the longest eruption periods—particularly second premolars and second molars—a margin of ±1.5 years should be applied.

## 4. Discussion

This study presents the first up-to-date reference standards for permanent-tooth emergence in Hungarian children.

Consistent with most international studies [22,23,24,25,29,30,31,32,33,34], we found that girls experience earlier permanent-tooth emergence than boys, although exceptions exist (e.g., Korean [35] and Pakistani [36] studies). This pattern is typically attributed to the general developmental lead observed in females [37,38]. Statistically significant differences were found for all teeth except the mandibular central incisor, and this finding aligns with those from Europe and the Middle East [22,23].

The sequence of permanent-tooth emergence differed by sex. In boys, mandibular central incisors preceded the first molars, a pattern similar to findings in Czech boys [25], while in girls, the first molars erupted first. Emergence curves showed near-simultaneous eruption of these teeth in both sexes, consistent with the Jordanian study by Shaweesh [22].

Notably, in the mandibular supporting zone, boys showed an uncommon eruption sequence: the canine emerged later than the first premolar but earlier than the second premolar. Similar sequences have been reported in Lithuania [24], Jordan [22], and Malaysia [33]. Maxillary eruption sequences in boys resembled those from Central and Eastern Europe and the Middle East [22,23,24,25,26,39]. In contrast, the sequence observed in girls was more aligned with Western European patterns [29,30,31], as well as the pattern described in an Australian study [32].

A comparison with international median-based studies (Table 3 and Table 4) revealed general consistency with other Caucasian populations. However, we found variability in median emergence ages, ranging from 0.4 to 1.8 years across different countries. The largest deviations (>1 year) were seen in premolars and mandibular canines. Hungarian data closely matched Lithuanian [24] and Jordanian [22] findings but diverged from British data [31], suggesting possible genetic influences, as lifestyle differences in urbanized Europe are decreasing.

When compared with non-Caucasian populations, differences in both the onset and duration of eruption were more marked. African children show earlier emergence by 1–1.5 years and a shorter mixed-dentition period [40,41]. Asian children exhibit intermediate timing [33,35], while indigenous Australian children also display patterns of accelerated eruption [42]. These observations indicate that there are genetic and geographic components to dental development [16].

To ensure sample homogeneity, we excluded non-Caucasian subjects. The Hungarian emergence values thus provide relevant regional standards within the broader Caucasian context.

Mandibular teeth typically emerged earlier than their maxillary counterparts, although upper premolars erupted earlier than lower ones in boys—a less common pattern reported in several other populations [23,24,25,33,36]. In girls, in the eruption of premolars no significant inter-arch differences were found. Eruption symmetry between the left and right sides was confirmed, consistent with findings from most previous studies [22,24,25,32,33]. While some authors, such as Leroy [30], have reported minor asymmetries favoring one side, these differences were not statistically significant in our cohort. Lo and Moyers [43] noted that eruption is generally symmetrical, except in cases where premature loss of deciduous teeth -often due to caries- accelerates the eruption of their permanent successors. This symmetry is likely attributable to balanced endocrine influences, which regulate the development of contralateral teeth in a coordinated manner, similar to other bilaterally symmetrical organs.

Compared to international standards (e.g., AAPD Reference Manual [20], Proffit [21]), Hungarian children generally exhibited earlier eruption of several teeth, particularly the upper incisors, premolars, and second molars. These differences—often occurring up to 0.5–1 year—highlight the importance of using updated, population-specific standards.

The eruption timelines presented in the AAPD Reference Manual [20] are based on the work of Logan and Kronfeld [44], which have only undergone minor revisions since their original publication in 1933. These tables do not report median values but instead present the interval between the 25th and 75th percentiles as the standard eruption window, defining the physiological range as ±1 year. However, the analysis of eruption probability curves in the present study indicates that this uniform standard does not reflect the actual variability in eruption timing across different teeth. Ekstrand et al. [45] similarly demonstrated that the emergence period for first molars is at least one year shorter than that for second molars. For second premolars and second molars, a broader interval of ±1.5 years is required to encompass the range between the 5th and 95th percentiles, suggesting the need to revise current reference standards to accommodate tooth-specific variability.

The main limitations of this study relate to its cross-sectional design and the lack of radiographic verification or formal examiner calibration. However, the use of standardized recording methods, a large representative sample, and robust probit regression analysis support the reliability of our results. Despite being limited to an urban population, these data provide an essential baseline for future national studies.

Although formal intra- and inter-examiner calibration sessions were not performed prior to data collection, all participating pediatric dentists were trained specialists following identical recording protocols mandated by national school health regulations. The binary scoring system (tooth present or absent) minimized subjective interpretation, reducing the potential impact of inter-observer variability. Nonetheless, future studies should include calibration exercises and kappa reliability testing to ensure complete harmonization among examiners.

Since the study population consisted mainly of urban children, the findings may not fully represent rural populations, where differences in nutrition and socioeconomic factors can influence eruption timing.

Finally, due to the cross-sectional design, we were unable to assess the impact of premature loss of primary teeth on the timing of permanent tooth eruption.

Since our data align with findings from other Caucasian populations, but they diverge from older international standards, especially British and North American ones, they can provide provisional guidance for professionals throughout Central Europe, until data based on a longitudinal study and representing the entire population are available.

## 5. Conclusions

This study provides the first contemporary reference data for the emergence timing and sequence of permanent teeth in Hungarian children. Sex-specific percentile charts (5th–95th) were developed to aid clinicians in assessing eruption delays and identifying potential agenesis. The results show that many children have erupted permanent teeth before school entry, emphasizing the importance of initiating oral health prevention in kindergarten.

Hungarian children demonstrated eruption patterns broadly consistent with other Caucasian populations but notably earlier than those reported in older British and North American standards. These data thus offer an updated and regionally relevant benchmark for Central European clinicians.

Future longitudinal and nationwide studies are recommended to confirm these trends and to evaluate regional differences between urban and rural populations.

## Figures and Tables

**Table 1:** Median ages of permanent tooth emergence in Hungarian boys and girls (in months).

	Boys		Girls		Difference	
	Median	(95% CI)	Median	(95% CI)	Months	*p*-Value
Maxilla						
I1	85.7	(84.4–87.0)	82.1	(80.6–83.4)	3.6	<0.001
I2	98.0	(95.5–98.6)	93.5	(91.9–95.1)	4.5	<0.001
C	135.9	(134.4–137.5)	129.7	(128.1–131.3)	6.2	<0.001
P1	121.4	(120.1–122.7)	119.4	(118.1–120.8)	2.0	0.009
P2	133.1	(131.6–134.7)	131.2	(129.6–132.7)	1.9	0.017
M1	76.4	(74.4–78.2)	73.7	(71.6–75.6)	2.7	0.004
M2	150.2	(148.5–152.0)	146.2	(144.5–147.9)	4.0	<0.001
Mandible						
I1	74.8	(72.3–76.9)	72.6	(69.8–74.9)	2.2	NS
I2	88.7	(87.0–90.4)	85.2	(83.4–87.0)	3.5	<0.001
C	124.5	(122.5–126.9)	115.6	(114.2–117.2)	8.9	<0.001
P1	123.5	(121.4–126.2)	119.4	(117.6–121.7)	4.1	<0.001
P2	135.4	(133.9–136.9)	131.3	(129.9–132.8)	4.1	<0.001
M1	75.2	(72.8–77.3)	72.3	(69.8–74.5)	2.9	0.042
M2	144.3	(142.2–146.5)	141.6	(139.5–143.7)	2.7	0.002

I1—central incisor, I2—lateral incisor, C—canine, P1—first premolar, P2—second premolar, M1—first molar, M2—second molar; NS—not significant.

**Table 2:** Percentiles of ages at emergence by sex and for the total sample (in years).

	Boys					Girls					Total Sample						
	Percentile					Percentile					Percentile					Difference	
	5th	25th	50th Median	75th	95th	5th	25th	50th Median	75th	95th	5th	25th	50th Median	75th	95th	5–95	25–75
Maxilla																	
I1	6.03	6.69	7.14	7.60	8.25	5.73	6.38	6.84	7.30	7.95	5.84	6.52	6.99	7.46	8.14	2.30	0.94
I2	6.67	7.51	8.09	8.67	9.51	6.37	7.21	7.79	8.38	9.21	6.49	7.35	7.94	8.54	9.40	2.91	1.19
C	9.37	10.52	11.33	12.13	13.28	8.85	10.01	10.81	11.61	12.76	9.10	10.27	11.07	11.88	13.05	3.95	1.61
P1	8.08	9.28	10.12	10.95	12.16	7.91	9.12	9.95	10.79	11.99	8.00	9.20	10.04	10.88	12.08	4.08	1.68
P2	8.82	10.16	11.10	12.03	13.37	8.65	10.00	10.93	11.86	13.21	8.74	10.08	11.02	11.95	13.29	4.55	1.87
M1	4.92	5.77	6.37	6.96	7.81	4.70	5.55	6.14	6.73	7.58	4.80	5.66	6.25	6.85	7.70	2.90	1.19
M2	10.18	11.56	12.52	13.48	14.86	9.85	11.23	12.18	13.14	14.52	10.01	11.39	12.35	13.30	14.68	4.67	1.91
Mandible																	
I1	4.90	5.69	6.24	6.78	7.57	4.72	5.51	6.05	6.60	7.39	4.81	5.60	6.15	6.69	7.48	2.67	1.09
I2	6.06	6.85	7.40	7.94	8.73	5.77	6.56	7.10	7.65	8.44	5.88	6.69	7.25	7.81	8.62	2.74	1.12
C	8.86	9.75	10.37	11.00	11.89	8.12	9.01	9.63	10.26	11.15	8.40	9.36	10.03	10.70	11.67	3.27	1.34
P1	8.47	9.55	10.30	11.04	12.12	8.13	9.21	9.95	10.70	11.78	8.29	9.38	10.14	10.90	11.99	3.70	1.52
P2	8.97	10.33	11.28	12.23	13.60	8.63	10.00	10.95	11.90	13.26	8.80	10.17	11.12	12.07	13.43	4.63	1.90
M1	4.87	5.69	6.27	6.84	7.66	4.63	5.45	6.02	6.59	7.41	4.74	5.57	6.14	6.72	7.55	2.81	1.15
M2	9.63	11.05	12.03	13.01	14.42	9.41	10.82	11.80	12.78	14.19	9.53	10.93	11.91	12.89	14.29	4.76	1.96

I1—central incisor, I2—lateral incisor, C—canine, P1—first premolar, P2—second premolar, M1—first molar, M2—second molar.

**Table 3:** Comparison of the median emergence ages of permanent teeth in boys (in years).

	Caucasian									Asian		African
	HUN	CRO	LTU	CZE	FIN	BEL ^a^	GBR	JOR	AUS ^c^	MAS	KOR ^a^	UGA ^b^
	Present	[23]	[24]	[25]	[29]	[30]	[31]	[22]	[32]	[33]	[35]	[34]
Maxilla												
I1	7.1	7.5	6.9	7.0	6.8	7.1	7.4	7.3	7.4	7.2	7.4	6.3
I2	8.2	8.5	8.0	8.0	8.1	8.3	8.7	8.5	8.6	8.6	8.5	8.5
C	11.3	11.6	11.1	11.3	11.3	11.5	12.0	11.6	11.8	11.0	10.9	10.8
P1	10.1	10.3	9.9	9.5	10.9	10.7	11.2	10.5	11.3	9.5	9.7	9.6
P2	11.1	10.8	10.8	11.0	11.7	11.6	12.3	11.4	12.1	10.4	10.5	9.5
M1	6.4	6.8	6.4	6.9	6.3	6.3	6.8	6.4	6.7	6.4	6.6	6.4
M2	12.5	12.6	12.3	12.7	12.4	12.3	12.8	12.6	12.7	12.2	12.8	11.0
Mandible												
I1	6.2	6.6	6.1	6.4	6.0	6.3	6.6	6.5	6.6	6.4	6.5	6.5
I2	7.4	7.7	7.2	7.3	7.1	7.4	7.8	7.5	7.8	7.5	7.5	5.8
C	10.4	10.9	10.4	9.4	10.5	10.6	11.0	10.6	11.0	10.2	10.2	10.1
P1	10.3	10.6	10.1	10.0	10.7	10.7	11.2	10.5	11.2	9.9	10.0	10.0
P2	11.3	10.9	11.1	10.9	11.6	11.7	12.2	11.7	12.1	10.9	10.9	10.8
M1	6.3	6.6	6.2	6.5	6.2	6.3	6.8	6.2	6.6	6.0	6.1	6.0
M2	12.0	11.9	11.7	12.4	12.0	11.8	12.3	12.2	12.2	11.4	11.7	11.5

^a^ longitudinal study, ^b^ mean values, ^c^ mixed population with Caucasian predominance. HUN—Hungary, CRO—Croatia, LTU—Lithuania, CZE—Czech Republic, FIN—Finland, BEL—Belgium, GBR—Great Britain, JOR—Jordan, AUS—Australia, MAS—Malaysia, KOR—Korea, UGA—Uganda, I1—central incisor, I2—lateral incisor, C—canine, P1—first premolar, P2—second premolar, M1—first molar, M2—second molar.

**Table 4:** Comparison of the median emergence ages of permanent teeth in girls (in years).

	Caucasian									Asian		African
	HUN	CRO	LTU	CZE	FIN	BEL ^a^	GBR	JOR	AUS ^c^	MAS	KOR ^a^	UGA ^b^
	Present	[23]	[24]	[25]	[29]	[30]	[31]	[22]	[31]	[33]	[35]	[34]
Maxilla												
I1	6.8	7.2	6.8	6.9	6.8	6.9	7.2	7.1	7.2	7.1	7.2	6.2
I2	7.8	8.3	7.6	7.6	7.6	7.9	8.2	8.1	8.2	8.5	8.0	7.2
C	10.8	11.1	10.5	10.4	10.8	11.0	11.4	11.1	11.2	10.5	10.0	9.3
P1	10.0	10.1	9.5	9.4	10.3	10.4	10.9	10.0	10.8	9.2	9.2	9.3
P2	10.9	10.7	10.6	10.9	11.6	11.4	11.8	11.0	11.7	10.2	10.1	10.1
M1	6.1	6.9	6.3	6.6	6.1	6.2	6.5	6.2	6.5	6.2	6.4	5.3
M2	12.2	12.4	12.1	12.5	11.9	12.0	12.4	12.3	12.3	12.0	12.1	10.7
Mandible												
I1	6.1	7.2	5.9	6.2	5.9	6.2	6.4	6.3	6.3	6.3	6.1	5.6
I2	7.1	7.2	6.9	7.2	6.8	7.1	7.4	7.3	7.4	7.3	7.2	6.8
C	9.6	10.0	9.6	9.1	9.7	9.7	10.3	9.8	10.1	9.5	9.4	9.7
P1	10.0	10.4	9.7	9.7	10.3	10.3	10.7	10.1	10.6	9.7	9.6	9.2
P2	10.9	10.9	10.6	10.6	11.3	11.4	11.9	11.2	11.7	10.6	10.5	10.2
M1	6.0	7.0	6.0	6.2	6.1	6.2	6.5	6.1	6.3	6.0	6.1	5.2
M2	11.8	11.8	11.3	11.8	11.6	11.6	12.0	11.7	11.8	11.0	11.3	10.3

^a^ longitudinal study, ^b^ mean values, ^c^ mixed population with Caucasian predominance. HUN—Hungary, CRO—Croatia, LTU—Lithuania, CZE—Czech Republic, FIN—Finland, BEL—Belgium, GBR—Great Britain, JOR—Jordan, AUS—Australia, MAS—Malaysia, KOR—Korea, UGA—Uganda, I1—central incisor, I2—lateral incisor, C—canine, P1—first premolar, P2—second premolar, M1—first molar, M2—second molar.

## Data Availability

The original contributions presented in this study are included in the article. Further inquiries can be directed to the corresponding author.
